# Enhanced Thermoelectric Performance of *β*-Ag_2_Se/RGO Composites Synthesized by Cold Sintering Process for Ambient Energy Harvesting

**DOI:** 10.3390/nano15211631

**Published:** 2025-10-26

**Authors:** Dulyawich Palaporn, Ikhwan Darmawan, Piyawat Piyasin, Supree Pinitsoontorn

**Affiliations:** 1 Department of Physics, Faculty of Science, Khon Kaen University, Khon Kaen 40002, Thailand; dulayawit.p@kkumail.com (D.P.);; 2 Grraduate School of Pure and Applied Sciences, University of Tsukuba, Tsukuba 305-8571, Ibaraki, Japan; 3 Global Zero Emission Research Center, National Institute of Advanced Industrial Science and Technology (AIST), Tsukuba 305-8569, Ibaraki, Japan; 4 Institution of Nanomaterials Research and Innovation for Energy, Faculty of Science, Khon Kaen University, Khon Kaen 40002, Thailand

**Keywords:** silver selenide, graphene, thermoelectric material, cold sintering process

## Abstract

Silver selenide (Ag_2_Se) is a promising *n*-type thermoelectric material for near-room-temperature energy harvesting due to its high electrical conductivity and low lattice thermal conductivity. In this study, Ag_2_Se-based composites were synthesized using a cold sintering process (CSP), enabling densification at low temperature under applied pressure. Reduced graphene oxide (RGO) was incorporated into the Ag_2_Se matrix in small amounts (0.25–1.0 wt.%) to enhance thermoelectric performance. Structural analysis confirmed phase-pure *β*-Ag_2_Se, while SEM and TEM revealed homogeneous RGO dispersion and strong interfacial adhesion. RGO addition led to a reduced carrier concentration due to carrier trapping by oxygen-bearing functional groups, resulting in decreased electrical conductivity. However, the absolute Seebeck coefficient increased with RGO content, maintaining a balanced power factor. Simultaneously, RGO suppressed thermal conductivity to below 0.75 W m^−1^ K^−1^ at room temperature. The optimal composition, 0.75 wt.% RGO, exhibited the highest average *zT* of 0.98 across the temperature range from room temperature to 383 K. These results demonstrate that combining the CSP with RGO incorporation offers a scalable and cost-effective strategy for enhancing the thermoelectric performance of Ag_2_Se-based materials.

## 1. Introduction

In recent years, green energy technologies have gained significant attention as a means to support global sustainable development [1]. Traditional reliance on fossil fuels faces growing challenges due to resource depletion and environmental concerns. As a result, various strategies have emerged to generate clean power. Among them, thermoelectric technology enables direct conversion of heat into electrical energy through solid-state devices [2]. Thermoelectric generators can operate when a temperature gradient is established across a material, making them attractive for low-grade heat recovery and decentralized power generation. The efficiency of thermoelectric materials is quantified by the dimensionless figure-of-merit (*zT*), defined as:

(1) $$zT=\frac{S^{2}\sigma }{\kappa }T$$

where *S* is the Seebeck coefficient, *σ* is the electrical conductivity, *T* is the absolute temperature, and *κ* is the thermal conductivity [3]. The product *S^2^σ* is referred to as the power factor (*PF*), which reflects the material’s ability to convert thermal energy into electrical power. Enhancing *zT* requires a delicate balance between these interdependent parameters.

Over the past decades, Bi_2_Te_3_-based thermoelectric materials have been widely recognized as the most efficient candidates for near-room-temperature applications [4]. However, tellurium (Te) is an extremely scarce element and its price is expected to rise sharply if Te-based materials reach mass-market adoption [5]. As a result, Te-free alternatives are being actively explored.

Silver selenide (Ag_2_Se) has emerged as a promising substitute for Bi_2_Te_3_ due to its high electrical conductivity, low lattice thermal conductivity, and narrow bandgap (~0.07–0.20 eV) [6,7,8]. Below 407 K, Ag_2_Se adopts an orthorhombic *β*-phase, which exhibits excellent thermoelectric properties. To further enhance its thermoelectric performance, various strategies have been employed, including precise stoichiometric control [9], doping [10], and nanostructuring [11].

Incorporating carbon-based materials has shown considerable potential for improving thermoelectric properties. Various carbon-based fillers, such as carbon nanotubes (CNTs) [12], multi-walled carbon nanotubes (MWCNTs) [13], and carbon black (CB) [14], have been explored to enhance electrical conductivity and phonon scattering. Among these, reduced graphene oxide (RGO) stands out due to its large surface area, which promotes uniform dispersion within the thermoelectric matrix and facilitates carrier concentration manipulation. Additionally, the high interface density introduced by RGO increases phonon scattering, effectively reducing thermal conductivity and improving *zT* [15,16]. For example, the integration of RGO into BiSbTe raised its *zT* from 1.09 to 1.29 at 300 K [17], while Thanh et al. reported a ~6.5-fold enhancement in *zT* for La/Nb-doped SrTiO_3_ upon RGO incorporation [18].

A recent study reported that incorporating 30 wt.% RGO into Ag_2_Se via hydrothermal synthesis followed by hot-pressing significantly reduced the thermal conductivity to ~0.492 W/mK at 333 K, achieving a *zT* of ~0.39 [15]. However, such a high RGO content compromises the mechanical integrity of the composite, as excessive filler disrupts the connectivity between Ag_2_Se grains, leading to reduced structural robustness. Moreover, the literature suggests that excessive RGO may suppress *zT* due to its tendency to attract charge carriers, thereby diminishing carrier mobility [19,20]. To address this, several studies have explored the use of low RGO concentrations to enhance the thermoelectric performance [21,22]. A small amount of RGO can effectively reduce thermal conductivity [23] while still maintaining sufficient carrier transport pathways [24,25]. This balance is crucial for optimizing *zT* without sacrificing mechanical or electrical integrity.

To consolidate thermoelectric materials, conventional sintering techniques such as spark plasma sintering (SPS) [26,27] and hot-pressing (HP) [28,29] are commonly employed. However, these methods require high sintering temperatures, which can induce grain growth, cause atomic diffusion, and degrade thermoelectric performance [30,31]. To overcome this limitation, the cold sintering process (CSP) has gained significant interest as a low-temperature densification technique [32]. The CSP utilizes a solvent to partially dissolve the target material, enabling particle rearrangement and densification at substantially reduced sintering temperatures. The solvent-assisted densification mechanism involves void filling between particles. Upon solvent evaporation, the dissolved material precipitates into these spaces, forming a dense microstructure [33,34]. A few thermoelectric materials have been consolidated using the CSP, for instance, Ca_3_Co_4_O_9_ [35], Bi_2_Te_3_ [36], and Cu_2_Se [37]. Moreover, the CSP can be modified to incorporate dopants or composite materials by introducing them in liquid form—such as solutions or colloids—during sintering [8,38]. In a previous study, AgNO_3_ solution was used as a Ag source to dope extra Ag^+^ into a Ag_2_Se system via the CSP, yielding highly dense samples with homogeneously distributed Ag dopants [39]. These findings demonstrate that the CSP offers a versatile and energy-efficient route for tailoring microstructure and thermoelectric properties of the materials by selecting appropriate solvents and processing conditions [40].

In this work, we leverage the advantages of the CSP to improve the dispersion of RGO within the Ag_2_Se matrix, enabling a substantial reduction in the required RGO content while maintaining high thermoelectric performance. The use of a liquid-phase RGO colloid ensures uniform distribution throughout the matrix, minimizing agglomeration and maximizing interfacial phonon scattering. Furthermore, the low sintering temperature inherent to the CSP preserves the nanostructure of Ag_2_Se and prevents RGO decomposition, eliminating the need for an inert atmosphere during processing. Consequently, a minimal RGO addition of just 0.75 wt.% was sufficient to significantly improve the thermoelectric properties of Ag_2_Se, enhancing electrical performance without compromising structural integrity. These findings highlight the potential of this method for future energy-harvesting applications, particularly in low-temperature environments.

## 2. Materials and Methods

### 2.1. Materials

Silver selenide (Ag_2_Se) powder was synthesized from elemental silver powder (Ag, 99.8% purity, Sigma-Aldrich, Saint Louis, MO, USA) and selenium powder (Se, 99.5% purity, Sigma-Aldrich, Saint Louis, MO, USA) without further purification. Reduced graphene oxide (RGO) was prepared according to our previous protocol [41].

### 2.2. Synthesis

Ag_2_Se powder was fabricated via a wet ball-milling method. Ag and Se powders were mixed in a stoichiometric ratio corresponding to Ag_2_Se. Yttria-stabilized zirconia (YSZ) balls with a diameter of 2 mm were loaded in a polypropylene vessel at a ball-to-powder mass ratio of 20:1, along with 15 mL of n-heptane as a milling medium. The mixture was milled at 250 rpm for 1 h. The resulting wet powder was then dried in an oven at 353 K for 24 h to obtain dry Ag_2_Se powder.

**Figure 1:**
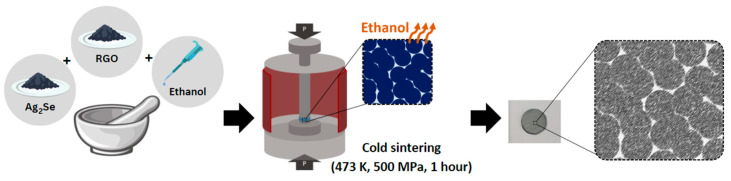
Schematic illustration for the fabrication of Ag_2_Se/RGO composites.

To prepare composite pellets, Ag_2_Se and RGO powder were manually mixed in a mortar, with RGO contents of 0.25, 0.50, 0.75, and 1.0 wt.%. Additionally, 0.1 mL of ethanol was added to facilitate mixing, and the blend was hand-grounded for 15 min. The mixture was then loaded into a stainless-steel mold and subjected to the cold sintering process (CSP) at 473 K under a uniaxial pressure of 500 MPa for 1 h in ambient atmosphere. A schematic illustration of the pellet fabrication process is shown in Figure 1, and the sample designations are summarized in Table 1.

### 2.3. Characterization

The sample density (*d*) was determined using the Archimedes principle. Crystal structure analysis was performed via X-ray diffraction (XRD, PANanalytical, EMPYREAN, Almelo, Netherlands) using Cu K_α_ radiation (λ = 1.54 Å). Microstructural features and elemental composition were examined using scanning electron microscopy (SEM, FEI, Helios NanoLab G3 CX, Waltham, MA, USA) coupled with energy dispersive X-ray spectroscopy (EDS). Transmission electron microscopy (TEM, Thermo Scientific, Talos F200X G2, Waltham, MA, USA) was used to investigate detailed nanostructure lattice planes in the samples. Thermoelectric properties including Seebeck coefficient and electrical conductivity were measured using a 4-point probe method (LSR-3, Linseis, Selb, Germany) over the temperature range from room temperature to 383 K under He atmosphere. Thermal diffusivity (*α*) was measured using a laser flash method (Linseis, LFA-500, Selb, Germany) under Ar atmosphere across the same temperature range. Thermal conductivity (*κ*) was calculated using the relation: *κ = αC_p_d*, where *C_p_* is the specific heat capacity, measured by differential scanning calorimetry (DSC, Rigaku, Thermo plus evo2, Tokyo, Japan). All thermoelectric measurements have been repeated three times at each temperature point to ensure reproducibility and accuracy.

## 3. Results and Discussion

### 3.1. Physical and Microstructural Analysis

**Figure 2:**
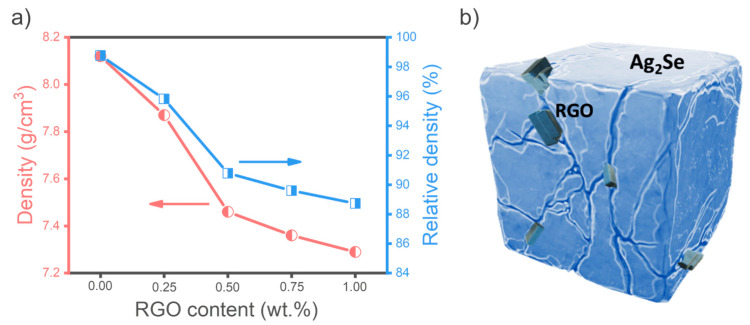
(**a**) Densities of Ag_2_Se/RGO samples with varying RGO contents; (**b**) Schematic illustration of RGO flakes dispersed in the Ag_2_Se-based matrix.

The relative density of the samples decreased progressively with increasing RGO content, as shown in Figure 2a. The pristine Ag_2_Se sample exhibited the highest density (98.8%), while the sample containing 1 wt.% RGO (Ag_2_Se–1%) showed the lowest density (88.7%). This trend is consistent with previous reports involving the incorporation of graphene-based materials into thermoelectric materials, such as Bi_2_Te_3_ [16], Cu_2_SnSe_3_ [42], and CoVSn [43], where increasing graphene content led to a reduction in bulk density. The observed decrease in density can be attributed to the significantly lower intrinsic density of RGO compared to Ag_2_Se. Furthermore, RGO flakes may adhere between Ag_2_Se grains, impeding particle compaction and introducing voids between grains, as schematically illustrated in Figure 2b. These microstructural disruptions contribute to the overall reduction in sample density and confirm the successful incorporation of RGO into the Ag_2_Se matrix.

**Figure 3:**
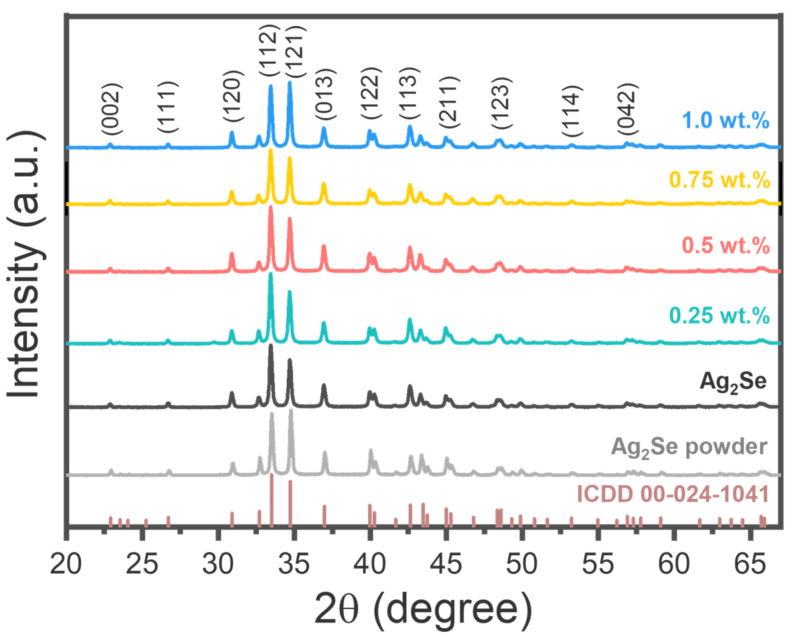
XRD patterns of Ag_2_Se samples and Ag_2_Se/RGO composites along with the *β*-Ag_2_Se reference pattern.

The crystallographic structure of the samples was examined using XRD, as shown in Figure 3. The diffraction patterns match well with the ICDD reference (00-024-1041), confirming the presence of *β*-Ag_2_Se with an orthorhombic structure and space group p2_1_2_1_2_1_. Rietveld refinement results, summarized in Table S1 yielded lattice parameters of *a* = 4.34 Å, *b* = 7.07 Å, and *c* = 7.77 Å—characteristic of the orthorhombic *β*-Ag_2_Se phase. These values are consistent with previous reports [44,45]. Importantly, the orthorhombic crystal structure of *β*-Ag_2_Se remained stable upon RGO incorporation, indicating that the compositing process did not significantly alter the crystallographic parameters. However, no distinct diffraction peaks corresponding to RGO were observed in any of the composite samples. This absence is attributed to the relatively low-crystalline RGO compared to the highly crystalline Ag_2_Se matrix, which dominates the diffraction signal and masks the presence of RGO. Therefore, to confirm the successful incorporation of RGO within the Ag_2_Se matrix, morphological analysis was employed as a complementary approach.

**Figure 4:**
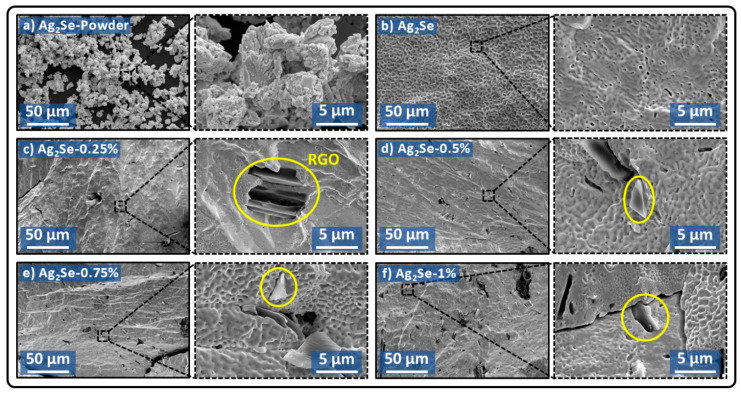
SEM images at low and high magnifications of (**a**) Ag_2_Se powder; (**b**) pristine Ag_2_Se sample, (**c**–**f**) Ag_2_Se composites with 0.25–1.0 wt.% RGO. The yellow circles highlight the RGO flaks adhered to Ag_2_Se grains.

SEM analysis was conducted to study the surface morphology of the Ag_2_Se-based samples, as shown in Figure 4. In Figure 4a, the pristine Ag_2_Se powder exhibits particle sizes ranging from approximately 3 to 10 µm in low-magnification images. Higher-magnification observations reveal that these micro-sized particles are composed of smaller, agglomerated primary particles. In addition, Figure 4b–f display the microstructure of samples consolidated via the CSP with varying RGO contents (0–1.0 wt.%). The pristine sample (Figure 4b) shows a densely packed morphology, consistent with its highest relative density of 98.8%. In contrast, the adherence of RGO to the Ag_2_Se matrix is clearly observed in Figure 4c–f. Low-magnification images reveal a progressive increase in RGO dispersion with rising nominal composition, indicating that the CSP effectively facilitates homogeneous integration of RGO within the matrix. At higher magnification, the RGO sheets are observed directly attached to Ag_2_Se grains. However, at elevated RGO concentrations (1.0 wt.%), the presence of RGO appears to disrupt particle packing, leading to microstructural discontinuities and a more fractured matrix (Figure 4f). These morphological changes further support the observed reduction in sample density with increasing RGO content.

To complement the morphological analysis, EDS was performed on all samples, with the results presented in Figure S1 The atomic ratio of silver to selenium remains close to the stoichiometric 2:1 ratio, confirming the chemical integrity of Ag_2_Se. In addition, carbon and oxygen signals were detected, with increasing intensity correlating with higher RGO content. This is consistent with the composition of RGO, which primarily consists of carbon and contains oxygen-bearing functional groups. These findings confirm the successful incorporation of RGO into the Ag_2_Se matrix via the CSP.

**Figure 5:**
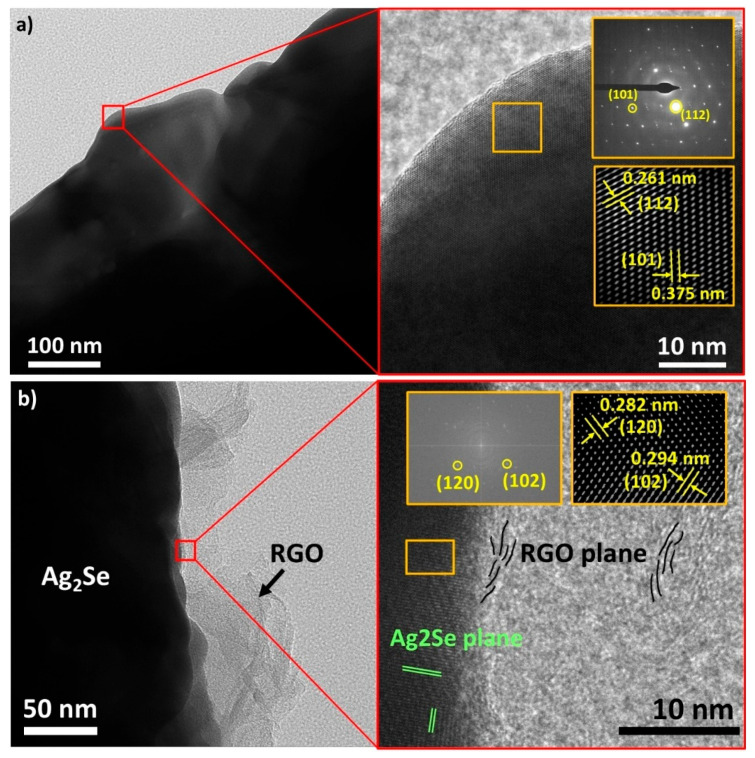
TEM images at low and high magnifications of (**a**) pristine Ag_2_Se sample; (**b**) Ag_2_Se/RGO composites.

To investigate the nanostructure of the prepared samples in greater detail, TEM analysis was performed on both pristine and RGO-composited Ag_2_Se samples. As shown in Figure 5a, the low-magnification image of the pristine Ag_2_Se sample reveals a densely consolidated microstructure, confirming that the CSP is effective in densifying the powders into a compact bulk sample. Furthermore, no residual liquid phase originating from the CSP was observed, indicating that the consolidation was successfully achieved without leaving unwanted secondary phases. The red square highlights an area selected for high-resolution analysis. The selected area electron diffraction (SAED) pattern (top inset) displays sharp diffraction spots of the orthorhombic *β*-Ag_2_Se phase, indicating a highly ordered crystal structure. Fast Fourier transform (FFT) analysis of the high-resolution TEM image (bottom inset) confirms the presence of the (112) and (101) planes, with interplanar spacings of 0.261 nm and 0.375 nm, respectively, consistent with the XRD results. In contrast, Figure 5b presents the TEM image of an RGO-composited Ag_2_Se sample. At low magnification, RGO sheets are clearly seen adhering to the surface of Ag_2_Se particles, confirming successful compositing via the CSP. Upon closer examination, lattice fringes corresponding to the Ag_2_Se phase remain visible, although the presence of RGO introduces localized irregularities in the lattice. The inset (yellow square) identifies the (120) and (102) planes of Ag_2_Se with interplanar spacings of 0.282 nm and 0.294 nm, respectively. Meanwhile, the RGO region exhibits partial crystallinity, but with poor orientation and less distinct lattice ordering.

### 3.2. Transport and Thermoelectric Properties

**Figure 6:**
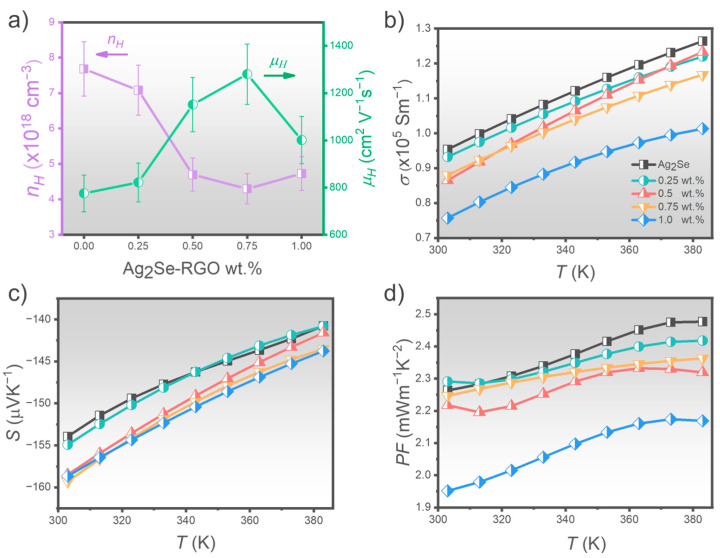
(**a**) Carrier concentration and mobility of the Ag_2_Se-based samples with varying RGO from 0 to 1.0 wt.%, Temperature-dependent; (**b**) electrical conductivity (*σ*); (**c**) Seebeck coefficient; and (**d**) power factor (*PF*) of the Ag_2_Se-based samples with varying RGO contents.

Carrier concentration (*n_H_*) and electron mobility (*μ_H_*) were measured by using the Hall effect technique, as shown in Figure 6a. The pristine Ag_2_Se sample exhibited the highest *n_H_* (~7.68 × 10^18^ cm^−3^), which gradually decreased to 7.08 × 10^18^ cm^−3^ at 0.25 wt.% RGO. Beyond 0.5 wt.% RGO, *n_H_* further declined and stabilized around 4.50 × 10^18^ cm^−3^. On the other hand, *μ_H_* displayed an inverse trend. The pristine sample showed a mobility of 775 cm^2^ V^−1^ s^−1^, which increased with RGO content, peaking at 1280 cm^2^ V^−1^ s^−1^ for the 0.75 wt.% sample. However, at 1.0 wt.% RGO, *μ_H_* dropped sharply to 1001 cm^2^ V^−1^ s^−1^.

The reduction in *n_H_* is attributed to the presence of oxygen-bearing functional groups on RGO, which act as carrier traps by attracting free electrons—effectively lowering the carrier concentration. This carrier-trapping mechanism has been previously reported thermoelectric systems containing RGO, for instance, in the Bi-Sb-Te/RGO [46] and SnSe/RGO [47] systems. However, at higher RGO loadings (>0.5 wt.%), the tendency of RGO to agglomerate into larger clusters reduces its interfacial contact with the Ag_2_Se matrix, thereby limiting further carrier trapping. Meanwhile, the extended surface area of RGO facilitates efficient charge transport by minimizing grain boundary scattering, which enhances *μ_H_* [48,49]. Nevertheless, the abrupt decline in mobility at 1.0 wt.% RGO is likely due to structural fractures observed in the sample (Figure 4f), which disrupt carrier pathways. This highlights that excessive filler content can compromise structural integrity and hinder charge transport.

Figure 6b illustrates the temperature-dependent electrical conductivity (*σ)* of Ag_2_Se samples with varying RGO content. A slight reduction in *σ* is observed with initial RGO addition, followed by a more pronounced decline at higher concentrations. Specifically, electrical conductivity decreases from 9.5 × 10^4^ S/m for pristine Ag_2_Se to 9.3, 8.6, 8.8, and 7.6 × 10^4^ S/m for samples containing 0.25, 0.5, 0.75 and 1.0 wt.% RGO, respectively. This trend indicates that the reduction in *σ* for 0.25–0.75 wt.% RGO samples primarily stems from a decrease in *n_H_*, which is directly proportional to electrical conductivity according to the relation:

(2) $$\sigma =n_{H}e\mu_{H}$$

where *e* is the electron charge (1.602 × 10^−19^ C). Although *μ_H_* increases with RGO content, the dominant influence of decreasing *n_H_* results in an overall decline in *σ*. At 1.0 wt.% RGO, *n_H_* stabilizes relative to 0.5 and 0.75 wt.% samples. Nevertheless, electrical conductivity continues to decrease. This behavior is attributed to the fractured microstructure of the Ag_2_Se matrix, as shown in Figure 4f, which disrupts electron transport pathway. The corresponding reduction in *μ_H_*, discussed earlier, further contributes to the significant drop in electrical conductivity to its lowest observed value.

The Seebeck coefficient (*S*) values are plotted in Figure 6c. The negative sign confirms that Ag_2_Se and its composites exhibit *n*-type thermoelectric behavior. Moreover, the absolute Seebeck coefficient (|*S*|) decreases with increasing temperature, indicating semiconductor-like behavior. For Ag_2_Se–0.25%, the Seebeck coefficient remains comparable to the pristine sample (|*S*|~154 μV K^−1^). However, with RGO content above 0.5 wt.%, |*S*| increases slightly to ~159 μV K^−1^ and then saturates. This enhancement in |*S*| upon RGO addition can be interpreted using the following relation [3]:

(3) $$S=\frac{8\pi^{2}{k_{B}}^{2}}{3eh^{2}}m^{*}T{\left[\frac{\pi }{3n}\right]}^{\frac{2}{3}}$$

where k_B_, m^*^, and *h* are the Boltzmann constant, the effective mass of charge carriers, and the Planck’s constant, respectively. As shown in Figure 6a, *n_H_* decreases with increasing RGO content up to 0.5 wt.%, which reasonably explains the gradual increase in |*S*| due to its inverse dependence on *n_H_*. Beyond 0.5 wt.%, *n_H_* tends to stabilize, resulting in a corresponding saturation of the Seebeck coefficient.

Figure 6d presents the power factor (*PF*) of the Ag_2_Se samples. Although the Seebeck coefficient improves with increasing RGO content, the enhancement is insufficient to offset the significant reduction in electrical conductivity. Consequently, *PF* slightly decreases from 2.4 to 2.3 mW m^−1^ K^−2^ as the RGO content increases from 0.25 to 0.75 wt.% at 383 K. A more pronounced drop in *PF* is observed for the 1.0 wt.% RGO sample, reaching 2.1 mW m^−1^ K^−2^. These results indicate that RGO incorporation affects the *n_H_*, leading to reduced electrical conductivity. While the increase in absolute Seebeck coefficient helps maintain a relatively balanced *PF* in the 0.25–0.75 wt.% range, further RGO addition to 1.0 wt.% adversely impacts the microstructure and charge transport, ultimately compromising the power factor of the composite.

**Figure 7:**
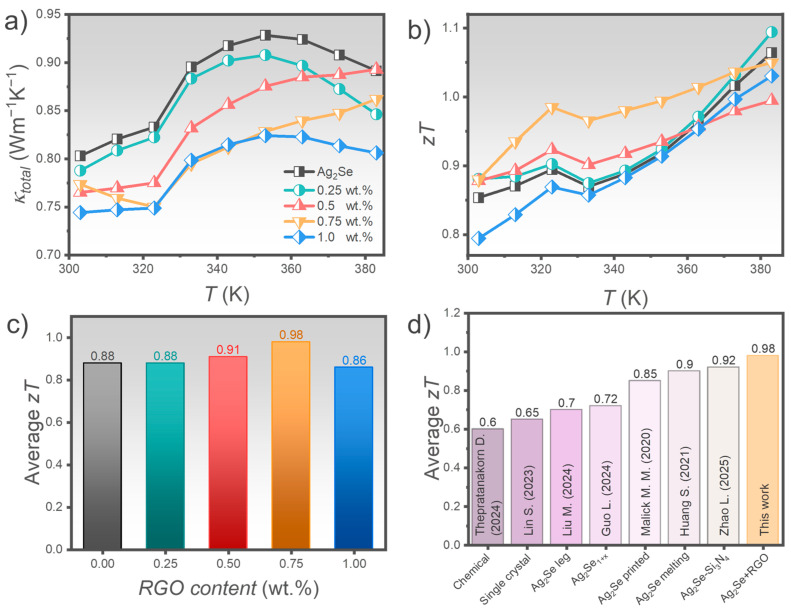
Thermoelectric performance of Ag_2_Se samples with varying RGO content: (**a**) temperature-dependent total thermal conductivity (*κ_total_*), (**b**) temperature-dependent *zT* values, (**c**) average *zT* values for each composition, (**d**) comparison of average *zT* with previously reported Ag_2_Se-based materials (Data from [9,39,50,51,52,53,54]).

Figure 7a depicts the total thermal conductivity (*κ_total_*) of Ag_2_Se samples with varying RGO content. The pristine sample exhibits maximum *κ_total_* of 0.93 W m^−1^ K^−1^ at 353 K. With increasing RGO content from 0.25 to 1.0 wt.%, *κ_total_* gradually decreases, with minimum values below 0.75 W m^−1^ K^−1^ at 303 K, indicating that RGO incorporation effectively suppresses thermal transport. This behavior can be interpreted by decomposing *κ_total_* into two components: electronic thermal conductivity (*κ_e_*) and lattice thermal conductivity (*κ_l_*), as shown in Figure S2. The electronic contribution is calculated using the Wiedemann–Franz law: *κ_e_ = LσT*, where *L* is the Lorentz number (1.77×10^−8^ WΩ K^−2^) [8]. As electrical conductivity decreases with increasing RGO content, *κ_e_* also declines (Figure S2a). In contrast, *κ_l_* exhibits a more complex trend. It slightly increases with the addition of 0.25 wt.% RGO (Figure S2b), likely due to RGO filling voids between Ag_2_Se particles and facilitating phonon transport. However, with further RGO addition (0.5–0.75 wt.%), *κ_l_* decreases, as excess RGO introduces additional interfacial boundaries, enhancing phonon scattering. Interestingly, the sample with 1.0 wt.% RGO shows a rise in *κ_l_*, attributed to RGO agglomeration forming continuous pathways for phonon transport. Given RGO possessing inherently high thermal conductivity, these regions reduce interfacial scattering and elevate *κ_l_*.

Figure 7b presents the temperature-dependent *zT*. Overall, *zT* improves with increasing RGO content up to 0.75 wt.%. The 0.25 wt.% RGO sample shows a modest enhancement over pristine Ag_2_Se, while the 0.5 and 0.75 wt.% samples exhibit more substantial improvement due to reduced thermal conductivity and relatively stable *PF*. Notably, the 0.75 wt.% RGO sample maintains a high *zT* (0.9–1.0) across the entire measured temperature range, demonstrating superior thermoelectric performance. In contrast, the 1.0 wt.% RGO sample shows the lowest *zT* due to a sharp decline in *PF*, despite its low thermal conductivity.

Although the 0.25 wt.% sample achieves the highest *zT* at 383 K, its performance is limited to a narrow temperature window. In contrast, the 0.75 wt.% RGO sample, with an average *zT* of 0.98 from room temperature to 383 K (Figure 7c), offers the best overall thermoelectric performance across the measured range, making it the optimal composition. Figure 7d compares the average *zT* values of Ag_2_Se-based materials from previous studies, showing that the *zT* achieved in this work surpasses previously reported values. These results demonstrate that the strategies employed in this study are effective, maintaining a reasonable *PF* while significantly reducing total thermal conductivity. Ultimately, the combination of the CSP technique and RGO incorporation into Ag_2_Se proves to be a promising approach for enhancing thermoelectric performance, offering clear advantages in terms of cost reduction and scalability for practical applications.

## 4. Conclusions

This work demonstrates that the CSP is an effective method for fabricating dense Ag_2_Se-based thermoelectric composites at low temperature under applied pressure. The incorporation of RGO into the Ag_2_Se matrix successfully enhances thermoelectric performance by reducing lattice thermal conductivity and increasing the Seebeck coefficient, which reached up to –159 μV K^−1^ for the 0.75 wt.% RGO sample. Although RGO addition decreases electrical conductivity from 9.5 × 10^4^ S m^−1^ for pristine Ag_2_Se to 7.6 × 10^4^ S m^−1^ at 1.0 wt.% RGO due to carrier trapping and microstructural disruption, the overall *PF* remains reasonable, ranging from 2.4 to 2.1 mW m^−1^ K^−2^ at 383 K across compositions. The optimal formulation, containing 0.75 wt.% RGO, achieves the highest average *zT* of 0.98 across the measured temperature range (303-383 K). Compared to previous strategies, this approach offers a practical and cost-efficient alternative for improving *n*-type thermoelectric materials. The synergy between the CSP and RGO incorporation provides a promising route for scalable energy-harvesting applications near room temperature.

## Figures and Tables

**Table 1:** Sample designation for the Ag_2_Se and Ag_2_Se/RGO samples.

Sample	RGO wt.%	Phase
Ag_2_Se-powder	-	Powder
Ag_2_Se	-	Pellet
Ag_2_Se-0.25%	0.25	Pellet
Ag_2_Se-0.5%	0.5	Pellet
Ag_2_Se-0.75%	0.75	Pellet
Ag_2_Se-1%	1.0	Pellet

## Data Availability

The original contributions presented in this study are included in the article. Further inquiries can be directed to the corresponding author(s).

## Supplementary Materials

The following supporting information can be downloaded at: https://www.mdpi.com/article/10.3390/nano15211631/s1, Figure S1: SEM images with corresponding EDS mapping for (a) Ag_2_Se powder, (b) pristine Ag_2_Se sample, (c–f) Ag_2_Se composites with 0.25–1.0 wt.% RGO; Figure S2: (a) electronic thermal conductivity (*κ_e_*) and (b) lattice thermal conductivity (*κ_l_*) of pristine Ag_2_Se and Ag_2_Se/RGO composite samples; Table S1: The crystal structural parameters of Ag_2_Se and Ag_2_Se/RGO composites, determined by Rietveld refinement of the XRD patterns.

## References

[1] Østergaard: P.A., Duic N., Noorollahi Y., Kalogirou S. (2023). Advances in renewable energy for sustainable development. Renew. Energy.

[2] Zhang X., Zhao L.-D. (2015). Thermoelectric materials: Energy conversion between heat and electricity. J. Mater..

[3] Snyder G.J., Toberer E.S. (2008). Complex thermoelectric materials. Nat. Mater..

[4] Mamur H., Bhuiyan M., Korkmaz F., Nil M. (2018). A review on bismuth telluride (Bi_2_Te_3_) nanostructure for thermoelectric applications. Renew. Sustain. Energy Rev..

[5] Guin S.N., Chatterjee A., Negi D.S., Datta R., Biswas K. (2013). High thermoelectric performance in tellurium free p-type AgSbSe_2_. Energy Environ. Sci..

[6] Ferhat M., Nagao J. (2000). Thermoelectric and transport properties of β-Ag_2_Se compounds. J. Appl. Phys..

[7] Ding Y., Qiu Y., Cai K., Yao Q., Chen S., Chen L., He J. (2019). High performance n-type Ag_2_Se film on nylon membrane for flexible thermoelectric power generator. Nat. Commun..

[8] Palaporn D., Pinitsoontorn S., Kurosaki K., Snyder G.J. (2023). Porous Ag_2_Se Fabricated by a Modified Cold Sintering Process with the Average *zT* Around Unity Near Room Temperature. Adv. Mater. Technol..

[9] Guo L., Lu X., Hou Y., Zhang X., Li R., Jin M., Lin S. (2024). Stoichiometric manipulation to enhance the thermoelectric and mechanical performance of Ag_2_Se_1+x_. Chem. Phys. Lett..

[10] Jood P., Ohta M. (2020). Temperature-Dependent Structural Variation and Cu Substitution in Thermoelectric Silver Selenide. ACS Appl. Energy Mater..

[11] Yang D., Su X., Meng F., Wang S., Yan Y., Yang J., He J., Zhang Q., Uher C., Kanatzidis M.G. (2017). Facile room temperature solventless synthesis of high thermoelectric performance Ag_2_Se *via* a dissociative adsorption reaction. J. Mater. Chem. A.

[12] Nunna R., Qiu P., Yin M., Chen H., Hanus R., Song Q., Zhang T., Chou M.-Y., Agne M.T., He J. (2017). Ultrahigh thermoelectric performance in Cu_2_Se-based hybrid materials with highly dispersed molecular CNTs. Energy Environ. Sci..

[13] Sondors R., Gavars D., Spalva E., Kons A., Lohmus R., Volkova M., Meija R., Andzane J. (2023). Synthesis and enhanced room-temperature thermoelectric properties of CuO–MWCNT hybrid nanostructured composites. Nanoscale Adv..

[14] Palaporn D., Iadrat P., Yurata T., Changtong C., Pinitsoontorn S. (2024). Enhanced thermoelectric properties of Ag2Se by manipulation in carrier concentration via acetylene carbon black nanocomposites. Results Phys..

[15] Santhosh R., Abinaya R., Archana J., Ponnusamy S., Harish S., Navaneethan M. (2022). Controlled grain boundary interfaces of reduced graphene oxide in Ag_2_Se matrix for low lattice thermal conductivity and enhanced power factor for thermoelectric applications. J. Power Sources.

[16] Du Y., Li J., Xu J., Eklund P. (2019). Thermoelectric Properties of Reduced Graphene Oxide/Bi_2_Te_3_ Nanocomposites. Energies.

[17] Li C., Qin X., Li Y., Li D., Zhang J., Guo H., Xin H., Song C. (2016). Simultaneous increase in conductivity and phonon scattering in a graphene nanosheets/(Bi_2_Te_3_ )_0.2_(Sb_2_Te_3_)_0.8_ thermoelectric nanocomposite. J. Alloys Compd..

[18] Thanh T.T., Van Du N., Bae J., Choi S.Y., Ahmed T., Khan S.A., Cho J.Y., Nam W.H., Le D.D., Lee S. (2021). Combined effect of donor doping and RGO (reduced graphene oxide) coating in La/Nb-doped SrTiO_3_ thermoelectrics. Solid State Sci..

[19] Chueachot R., Nakhowong R. (2023). Achieving thermoelectric performance of rGO/Bi_0.5_Sb_1.5_Te_3_/Cu_2_Se_1–x_Te_x_ composites through the scattering engineering strategy. J. Materiomics..

[20] Wu C., Li J., Fan Y., Xing J., Gu H., Zhou Z., Lu X., Zhang Q., Wang L., Jiang W. (2019). The effect of reduced graphene oxide on microstructure and thermoelectric properties of Nb-doped A-site-deficient SrTiO_3_ ceramics. J. Alloys Compd..

[21] Okhay O., Tkach A. (2021). Impact of Graphene or Reduced Graphene Oxide on Performance of Thermoelectric Composites. C.

[22] Amollo T.A., Mola G.T., Kirui M.S.K., Nyamori V.O. (2017). Graphene for Thermoelectric Applications: Prospects and Challenges. Crit. Rev. Solid State Mater. Sci..

[23] Bark H., Ko M., Lee M., Lee W., Hong B., Lee H. (2018). Thermoelectric Properties of Thermally Reduced Graphene Oxide Observed by Tuning the Energy States. ACS Sustain. Chem. Eng..

[24] Rahman J.U., Van Du N., Nam W.H., Shin W.H., Lee K.H., Seo W.-S., Kim M.H., Lee S. (2019). Grain Boundary Interfaces Controlled by Reduced Graphene Oxide in Nonstoichiometric SrTiO_3-δ_ Thermoelectrics. Sci. Rep..

[25] Zhang K., Zhang Y., Wang S. (2013). Enhancing thermoelectric properties of organic composites through hierarchical nanostructures. Sci. Rep..

[26] Palaporn D., Kurosaki K., Pinitsoontorn S. (2023). Effect of Sintering Temperature on the Thermoelectric Properties of Ag_2_Se Fabricated by Spark Plasma Sintering with High Compression. Adv. Energy Sustain. Res..

[27] Chiu W.-T., Chen C.-L., Chen Y.-Y. (2016). A strategy to optimize the thermoelectric performance in a spark plasma sintering process. Sci. Rep..

[28] Palaporn D., Parse N., Tanusilp S.-A., Silpawilawan W., Kurosaki K., Pinitsoontorn S. (2020). Synthesis of Silicon and Higher Manganese Silicide Bulk Nano-composites and Their Thermoelectric Properties. J. Electron. Mater..

[29] Xu Y., Yan M., Jiang E., Zheng Z., Wang H., Duan B., Li G., Zhai P. (2023). Thermoelectric properties of Ag_2_Te prepared by one-step hot-pressing method. Mater. Lett..

[30] Namhongsa W., Omoto T., Fujii Y., Seetawan T., Kosuga A. (2017). Effect of the crystal structure on the electronic structure and electrical properties of thermoelectric GeSb_6_Te_10_ prepared by hot pressing. Scr. Mater..

[31] Delaizir G., Bernard-Granger G., Monnier J., Grodzki R., Kim-Hak O., Szkutnik P.-D., Soulier M., Saunier S., Goeuriot D., Rouleau O. (2012). A comparative study of Spark Plasma Sintering (SPS), Hot Isostatic Pressing (HIP) and microwaves sintering techniques on p-type Bi_2_Te_3_ thermoelectric properties. Mater. Res. Bull..

[32] Galotta A., Sglavo V.M. (2021). The cold sintering process: A review on processing features, densification mechanisms and perspectives. J. Eur. Ceram. Soc..

[33] Ndayishimiye A., Sengul M.Y., Sada T., Dursun S., Bang S.H., Grady Z.A., Tsuji K., Funahashi S., van Duin A.C., Randall C.A. (2020). Roadmap for densification in cold sintering: Chemical pathways. Open Ceram..

[34] Tee S.Y., Tan X.Y., Wang X., Lee C.J.J., Win K.Y., Ni X.P., Teo S.L., Seng D.H.L., Tanaka Y., Han M.-Y. (2022). Aqueous Synthesis, Doping, and Processing of n-Type Ag_2_Se for High Thermoelectric Performance at Near-Room-Temperature. Inorg. Chem..

[35] dos Santos A.M., Thomazini D., Gelfuso M.V. (2020). Cold sintering and thermoelectric properties of Ca_3_Co_4_O_9_ ceramics. Ceram. Int..

[36] Zhu B., Su X., Shu S., Luo Y., Tan X.Y., Sun J., Sun D., Zhang H., Zhang Q., Suwardi A. (2022). Cold-Sintered Bi_2_Te_3_-Based Materials for Engineering Nanograined Thermoelectrics. ACS Appl. Energy Mater..

[37] Piyasin P., Palaporn D., Kurosaki K., Pinitsoontorn S. (2024). High-performance thermoelectric properties of Cu_2_Se fabricated via cold sintering process. Solid State Sci..

[38] Jabr A., Jones H.N., Argüelles A.P., Trolier-McKinstry S., Randall C., Bermejo R. (2023). Scaling up the cold sintering process of ceramics. J. Eur. Ceram. Soc..

[39] Theprattanakorn D., Kaewmaraya T., Pinitsoontorn S. (2024). Boosting thermoelectric efficiency of Ag_2_Se through cold sintering process with Ag nano-precipitate formation. Int. J. Miner. Met. Mater..

[40] Kongsip N., Kaewmaraya T., Kamwanna T., Pinitsoontorn S. (2024). Enhancing thermoelectric properties of silver selenide through cold sintering process using aqua regia as a liquid medium. Next Mater..

[41] (2024). Synthesis, Characterization, and Electrochemical Properties of Reduced Graphene Oxide Produced from Sugarcane Bagasse for Supercapacitor Applications. Biointerface Res. Appl. Chem..

[42] Zhao D., Wang X., Wu D. (2017). Enhanced Thermoelectric Properties of Graphene/Cu_2_SnSe_3_ Composites. Crystals.

[43] Zaferani S.H., Ghomashchi R., Vashaee D. (2021). Assessment of Thermoelectric, Mechanical, and Microstructural Reinforcement Properties of Graphene-Mixed Heterostructures. ACS Appl. Energy Mater..

[44] Fang C., de Groot R., Wiegers G. (2002). Ab initio band structure calculations of the low-temperature phases of Ag_2_Se, Ag_2_Te and Ag_3_AuSe_2_. J. Phys. Chem. Solids.

[45] Rameshkumar S., Jaiganesh G., Jayalakshmi V., Palanivel B. (2015). Ab initio calculation of structural stability, electronic and optical properties of Ag_2_Se. AIP Conf. Proc..

[46] Shin W.H., Ahn K., Jeong M., Yoon J.S., Song J.M., Lee S., Seo W.S., Lim Y.S. (2017). Enhanced thermoelectric performance of reduced graphene oxide incorporated bismuth-antimony-telluride by lattice thermal conductivity reduction. J. Alloys Compd..

[47] Huang L., Lu J., Ma D., Ma C., Zhang B., Wang H., Wang G., Gregory D.H., Zhou X., Han G. (2019). Facile *in situ* solution synthesis of SnSe/rGO nanocomposites with enhanced thermoelectric performance. J. Mater. Chem. A.

[48] Bin Rahaman A., Sarkar A., Singha T., Chakraborty K., Dutta S., Pal T., Ghosh S., Datta P.K., Banerjee D. (2020). Electrical transport properties and ultrafast optical nonlinearity of rGO–metal chalcogenide ensembles. Nanoscale Adv..

[49] C A., Abinaya R., Mani N., Krishnan H.S. (2025). Strain Effect in the Layered MoS_2_-rGO Heterostructure with Enhanced Performance for Flexible Thermoelectric Applications. ACS Appl. Energy Mater..

[50] Lin S., Guo L., Wang X., Liu Y., Wu Y., Li R., Shao H., Jin M. (2023). Revealing the promising near-room-temperature thermoelectric performance in Ag_2_Se single crystals. J. Mater..

[51] Mallick M., Rösch A.G., Franke L., Gall A., Ahmad S., Gesswein H., Mazilkin A., Kuebel C., Lemmer U. (2020). New frontier in printed thermoelectrics: Formation of β-Ag_2_Se through thermally stimulated dissociative adsorption leads to high *ZT*. J. Mater. Chem. A.

[52] Huang S., Wei T.-R., Chen H., Xiao J., Zhu M., Zhao K., Shi X. (2021). Thermoelectric Ag_2_Se: Imperfection, Homogeneity, and Reproducibility. ACS Appl. Mater. Interfaces.

[53] Zhao L., Zhang H., Dong J., Zhang G., Cao Q., Ding Z., Wang S., Wang J., Li Z. (2025). Engineering Ag_2_Se thermoelectrics *via* amorphous nano-Si_3_N_4_: A dual-functional strategy for enhanced *zT* and mechanical strength. J. Mater. Chem. C.

[54] Liu M., Zhang X., Zhang S., Pei Y. (2024). Ag_2_Se as a tougher alternative to n-type Bi_2_Te_3_ thermoelectrics. Nat. Commun..

