# Supplementary material for: Enhanced Thermoelectric Performance of β-Ag2Se/RGO Composites Synthesized by Cold Sintering Process for Ambient Energy Harvesting
Source: Nanomaterials (Basel). 2025 Oct 26;15(21):1631. doi: 10.3390/nano15211631 (PMC12610702; doi:10.3390/nano15211631)
Supplement: Supplementary file 1 [file nanomaterials-15-01631-s001.zip › nanomaterials-3934924-supplementary.pdf]

nanomaterials

Supplementary Materials

Enhanced Thermoelectric Performance of β-Ag_2Se/RGO Composites Synthesized by Cold Sintering Process for Ambient Energy Harvesting

Dulyawich Palaporn [1], Ikhwan Darmawan [1,2], Piyawat Piyasin [1,3] and Supree Pinitsoontorn [1,4,*]

1 Department of Physics, Faculty of Science, Khon Kaen University, Khon Kaen, 40002, Thailand; dulayawit.p@kkumail.com (D.P.)
2 Grraduate School of Pure and Applied Sciences, University of Tsukuba, Tsukuba 305-8571, Ibaraki, Japan.
3 Global Zero Emission Research Center, National Institute of Advanced Industrial Science and Technology (AIST), Tsukuba 305-8569, Ibaraki, Japan.
4 Institution of Nanomaterials Research and Innovation for Energy, Faculty of Science, Khon Kaen University, Khon Kaen, 40002, Thailand
* Correspondence: psupree@kku.ac.th

Table S1. The crystal structural parameters of Ag_2Se and Ag_2Se/RGO composites, determined by Rietveld refinement of the XRD patterns.

Sample	a (Å)	b (Å)	c (Å)	2θ	FWHM	Size (nm)
Ag_2Se-powder	4.33	7.06	7.76	33.517	0.173	50.11
Ag_2Se	4.34	7.07	7.77	33.445	0.211	41.02
Ag_2Se-0.25%	4.34	7.07	7.77	33.448	0.207	41.83
Ag_2Se-0.5%	4.34	7.07	7.77	33.439	0.201	43.12
Ag_2Se-0.75%	4.34	7.07	7.77	33.447	0.189	45.82
Ag_2Se-1%	4.34	7.07	7.77	33.432	0.166	52.29

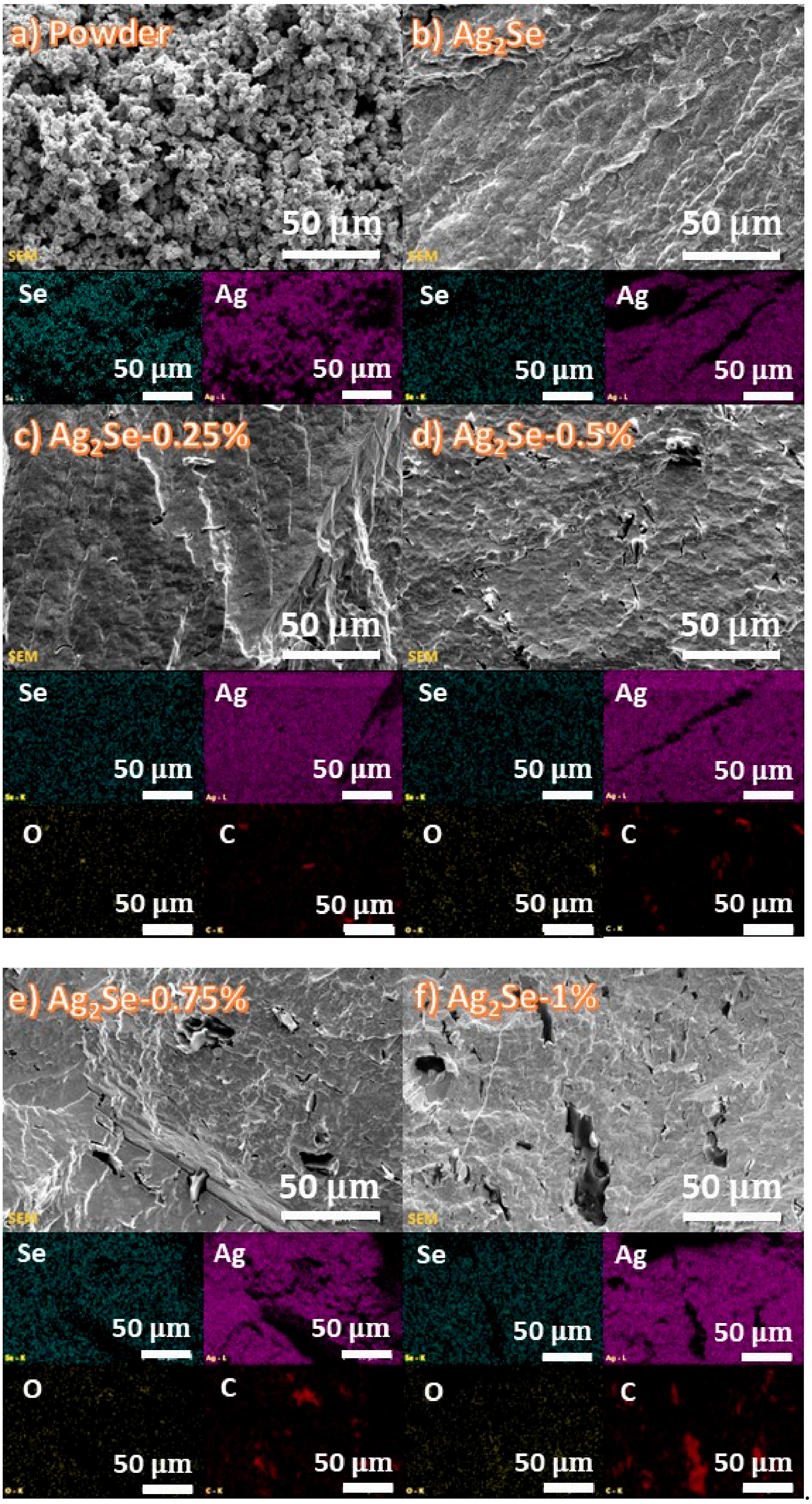

Figure S1. SEM images with corresponding EDS mapping for **a)** Ag_2Se powder, **b)** pristine Ag_2Se sample, **c)–f)** Ag_2Se composites with 0.25–1.0 wt.% RGO.

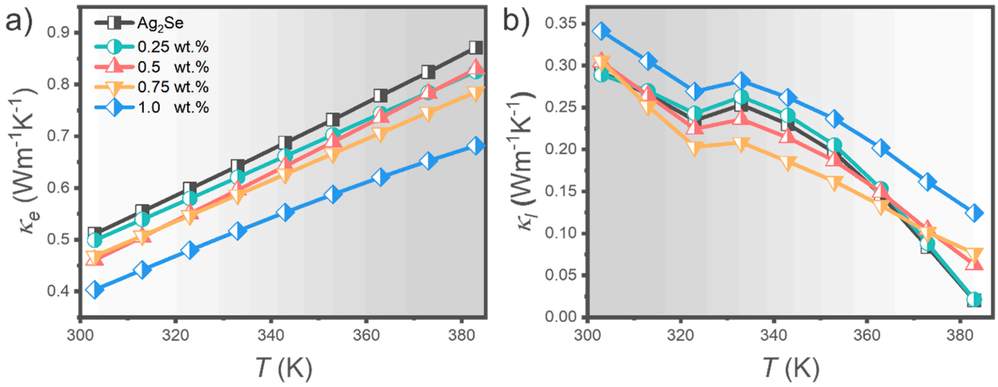

Figure S2. a) electronic thermal conductivity (κ_e) and **b)** lattice thermal conductivity (κ_l) of pristine Ag_2Se and Ag_2Se/RGO composite samples.
